# Automated quantification system predicts survival in rheumatoid arthritis–associated interstitial lung disease

**DOI:** 10.1093/rheumatology/keac184

**Published:** 2022-11-28

**Authors:** Ju Hyun Oh, Grace Hyun J. Kim, Gary Cross, Joseph Barnett, Joseph Jacob, Seokchan Hong, Jin Woo Song

**Affiliations:** 1 Department of Pulmonary and Critical Care Medicine, Asan Medical Center, University of Ulsan College of Medicine, Seoul, Republic of Korea; 2 Department of Radiological Sciences, David Geffen School of Medicine at UCLA, Los Angeles, USA; 3 Department of Radiology, Royal Free Hospital, Royal Free London NHS Foundation Trust, London, UK; 4 Department of Respiratory Medicine, University College London, London, UK; 5 Centre for Medical Image Computing, University College London, London, UK; 6 Department of Rheumatology, Asan Medical Center, University of Ulsan College of Medicine, Seoul, Republic of Korea

**Keywords:** interstitial lung disease, rheumatoid arthritis, fibrosis, prognosis, mortality

## Abstract

**Objective:**

The prognosis of rheumatoid arthritis–associated interstitial lung disease (RA-ILD) is difficult to predict because of the variable clinical course. This study aimed to determine the prognostic value of an automated quantification system (AQS) in RA-ILD.

**Methods:**

We retrospectively analysed the clinical data and high-resolution computed tomography (HRCT) images of 144 patients with RA-ILD. Quantitative lung fibrosis (QLF, sum of reticulation and traction bronchiectasis) and ILD (QILD; sum of QLF, honeycombing [QHC], and ground-glass opacity [QGG]) scores were measured using the AQS.

**Results:**

The mean age was 61.2 years, 43.8% of the patients were male, and the 5-year mortality rate was 30.5% (median follow-up, 52.2 months). Non-survivors showed older age, higher erythrocyte sedimentation rate (ESR), and greater AQS scores than survivors. In multivariable Cox analysis, higher QLF, QHC, and QILD scores were independent prognostic factors along with older age and higher ESR. In receiver-operating characteristic curve analysis, the QLF score showed better performance in predicting 5-year mortality than the QHC and QGG scores but was similar to the QILD score. Patients with high QLF scores (≥12% of total lung volume) showed higher 5-year mortality (50% vs. 17.4%, *P*<0.001) than those with low QLF scores and similar survival outcome to patients with idiopathic pulmonary fibrosis (IPF). Combining with clinical variables (age, ESR) further improved the performance of QLF score in predicting 5-year mortality.

**Conclusion:**

QLF scores might be useful for predicting prognosis in patients with RA-ILD. High QLF scores differentiate a poor prognostic phenotype similar to IPF.

## Introduction

Rheumatoid arthritis (RA) is an autoimmune disease associated with chronic inflammation, leading to the progressive destruction of multiple cartilages and bone. Interstitial lung disease (ILD) is the most common pulmonary complication of RA [1]. Clinically significant ILD occurs in approximately 10% of patients with RA and is one of the major causes of death [2, 3]. Although RA-associated ILD (RA-ILD) is generally known to have a better prognosis than idiopathic pulmonary fibrosis (IPF), the clinical course of RA-ILD is highly variable and difficult to predict [2]. In previous studies, several clinical variables such as older age, male sex, and a usual interstitial pneumonia (UIP) pattern on high-resolution computed tomography (HRCT) have been reported as predictors of mortality in patients with RA-ILD [4–8]. Lower lung function, especially decreased diffusing capacity of the lung for carbon monoxide (DLco) [9], and higher extent of fibrosis on HRCT were also reported to be useful predictors of prognosis in patients with RA-ILD [6, 8, 10]; however, the utility of these variables can be limited by insufficient patient effort or reader variability in interpreting imaging findings.

Recent studies have introduced computer-based analysis of HRCT imaging using techniques, such as the use of an automated quantification system (AQS), to more objectively evaluate the extent of fibrosis in patients with ILD [11–14]. Most AQS studies have been primarily conducted in patients with IPF or scleroderma-related ILD, but the roles of imaging parameters measured using the AQS in predicting prognosis in patients with RA-ILD are not well defined. The purpose of this study was to determine the prognostic value of HRCT parameters measured using an AQS in patients with RA-ILD.

## Methods

### Study population

A total of 158 patients with RA-ILD, who had baseline HRCT images at the time of ILD diagnosis at Asan Medical Center, Seoul, Republic of Korea, between November 1999 and July 2015 were screened for this study. Among them, 14 patients were excluded because of inadequate HRCT images (absence of volumetric images or thin section images) for AQS and visual assessments (n = 13) and lack of baseline lung function data (n = 1). Therefore, 144 patients with RA-ILD (biopsy-confirmed cases = 40, 27.8%) were finally included in this study. All patients met the RA diagnostic criteria of the American College of Rheumatology/European League Against Rheumatism [15], and the presence of ILD was confirmed on HRCT images. An IPF cohort, consisting of 159 patients diagnosed consecutively between January 2014 and July 2015 at Asan Medical Center, was included to compare prognosis with the RA-ILD cohort. All diagnoses of IPF were made through multidisciplinary discussions according to the American Thoracic Society (ATS) /European Respiratory Society (ERS)/Japanese Respiratory Society (JRS)/Latin American Thoracic Society (ALAT) IPF guidelines [16]. This research was conducted according to the guidelines of the Declaration of Helsinki, and approved by the Institutional Review Board of Asan Medical Center, Seoul, the Republic of Korea (approval no. 2020-0943). Patient consent was waived due to retrospective nature of this study.

### Data collection

The clinical and survival data of all patients were retrospectively collected from medical records, telephone interviews, or the records of the National Health Insurance of Korea. Spirometry parameters [17], DLco [18], and total lung capacity (TLC) determined with plethysmography [19] were measured according to previous recommendations and expressed as percentages of predicted values. All available clinical parameters were obtained within 3 months of initial HRCT date.

### Automated quantification of HRCT images

HRCT images were obtained, following standard protocols, at full inspiration without contrast enhancement. Details of computer-aided quantitative scoring system used in this study were described in previous reports [12, 20, 21]. Briefly, automated quantitative scoring of each HRCT image was established through five steps: 1) de-noising the image; 2) sampling each pixel from a grid; 3) converting the characteristics of grid intensities into texture features; 4) classifying the texture features of pixels as specific patterns, such as reticular pattern with architectural distortion, ground-glass opacity (GGO), or honeycombing using a built-in model; and 5) calculating the percentages of the classified pixels [12]. The quantitative lung fibrosis (QLF) (sum of reticulation and traction bronchiectasis), GGO (QGG), and honeycombing (QHC) scores were measured on HRCT using the AQS. The quantitative ILD (QILD) score (sum of QLF, QHC, and QGG) was also measured. Because of low prevalence in consolidation by visual, the automated quantification of consolidation was not included in this study. We applied the adaptive denoise based on the CT Hounsfield values to reduce the variation as descripted in previous studies [20, 21].

### Visual assessment of HRCT images

Visual assessment of HRCT images was performed by two radiologists (G.C. and J.B.) who were blinded to the patients’ information. The extents of GGO, reticulation, honeycombing and consolidation were semi-quantitatively scored on a lobar basis estimated to the nearest 5%. All computed tomography variables were expressed as a percentage of the total lung volume. The most disparate 5% (two standard deviations) of the values and any disagreement between the two radiologists were resolved by a third radiologist (J.J.). The ILD extent was defined as the sum of reticulation, honeycombing, and GGO. The HRCT patterns were classified according to the 2018 ATS /ERS/JRS/ALAT IPF guidelines [22]. A UIP pattern was defined as basal and subpleural predominant distribution of honeycombing, reticulation with or without traction bronchiectasis, and the absence of features such as consolidation, extensive GGOs, and mosaic attenuations to suggest alternative diagnosis [22]. The presence of emphysema was also evaluated [23].

### Statistical analysis

All values are expressed as means ± standard deviations for continuous variables or as percentages for categorical variables. Continuous variables were compared using the Student’s t-test or Mann–Whitney U-test, and categorical variables were compared using the chi-square test or Fisher’s exact test. The correlation between radiologist-determined scores and AQS scores was represented by the Pearson’s correlation coefficient, and the strength of the correlation was interpreted as follows: high (r ≥ 0.7), moderate (r = 0.5–0.7), and low (r < 0.5) [24]. Survival was evaluated using the Kaplan–Meier survival analysis and log-rank test. The follow-up period was calculated from the date of the initial HRCT to the date of death or time of censoring (date of vital status ascertainment: 31 Oct 2016) When performing the survival analysis, we set the criteria for censoring as follows: 1) survival at certain time point (5 years), 2) date of follow-up loss. We used a Cox proportional hazards model to identify risk factors for the mortality of RA-ILD. Variables with a *P*-value of < 0.1 in the unadjusted analysis were included in the multivariable analysis using backward elimination. The receiver-operating characteristic (ROC) curve analysis was used to evaluate the performance of the AQS scores in predicting mortality in patients with RA-ILD. Concordance statistics (C-statistics) were calculated to compare the performance of the prediction models. After selecting the best-performing model, each variable was assigned a point ranging from 0 to 2 according to coefficient values; that is, each Cox coefficient value was divided by the smallest Cox coefficient value, and the score was converted into an integer. Further, on a basis of the result of Fisher’s exact test or the chi-square test for the survival rate, the patients were classified into three stages according to the total points from 0 to 5 (stage I = 0–2, stage II = 3–4, stage III = 5). Internal validation using bootstrap was performed to control the concordance overestimate. All P-values were two-tailed, and statistical significance was set at *P* < 0.05. All statistical analyses were performed using SPSS software (version 21.0; IBM Corporation, Somers, NY, USA) and MedCalc Statistical Software (version 12.7.5; MedCalc Software bvba, Ostend, Belgium).

## Results

### Baseline characteristics

**Figure 1:**
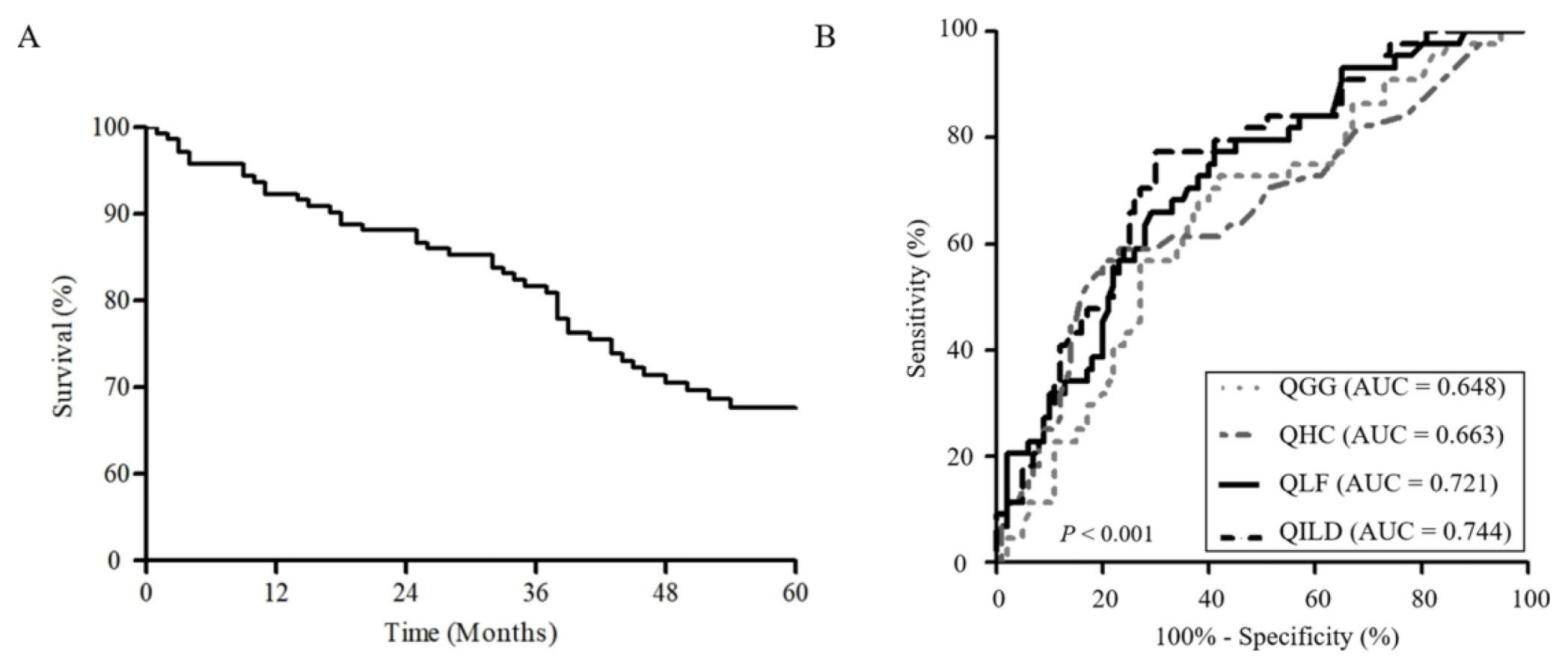
Survival outcomes of patients with RA-ILD and predictive performance of each AQS scores for 5-year mortality. A. Kaplan–Meier survival curves of total patients with RA-ILD, B. Comparison of receiver-operating characteristic curves for 5-year mortality among the AQS scores. RA-ILD, rheumatoid arthritis–associated interstitial lung disease RA-ILD, rheumatoid arthritis–associated interstitial lung disease; AQS, automated quantification system; QGG, quantification of ground-glass opacity; QHC, quantification of honeycombing; QLF, quantification of lung fibrosis; QILD, quantification of interstitial lung disease; AUC, area under the curve

The mean patient age was 61.2 years, and 43.8% of the patients were male (Table 1). The median follow-up period was 52.5 months (interquartile range [IQR], 36.2–90.5 months), and 44 (30.5%) patients died within 5 years of the diagnosis of ILD (Figure 1A). Of 144 patients, 121 patients (84.0%) received a diagnosis of ILD at a median of 53.0 months (IQR, 10.7-121.3 months) after receiving an RA diagnosis. In contrast, 11 (7.6%) had a diagnosis of ILD before being diagnosed with RA, with a median duration of 19.0 months (IQR, 12.0–48.0 months), and the remaining 12 patients were diagnosed concurrently. Non-survivors showed older age, more frequent ever-smoker, higher erythrocyte sedimentation rate (ESR), higher rheumatoid factor titre, and lower lung function (forced vital capacity [FVC], DLco, and TLC) than survivors (Table 1). Patients with IPF had older age and a higher proportion of men, but showed similar lung function (FVC and DLco) to patients with RA-ILD (Supplementary Table S1, available at *Rheumatology* online).

### Correlation of HRCT findings

**Figure 2:**
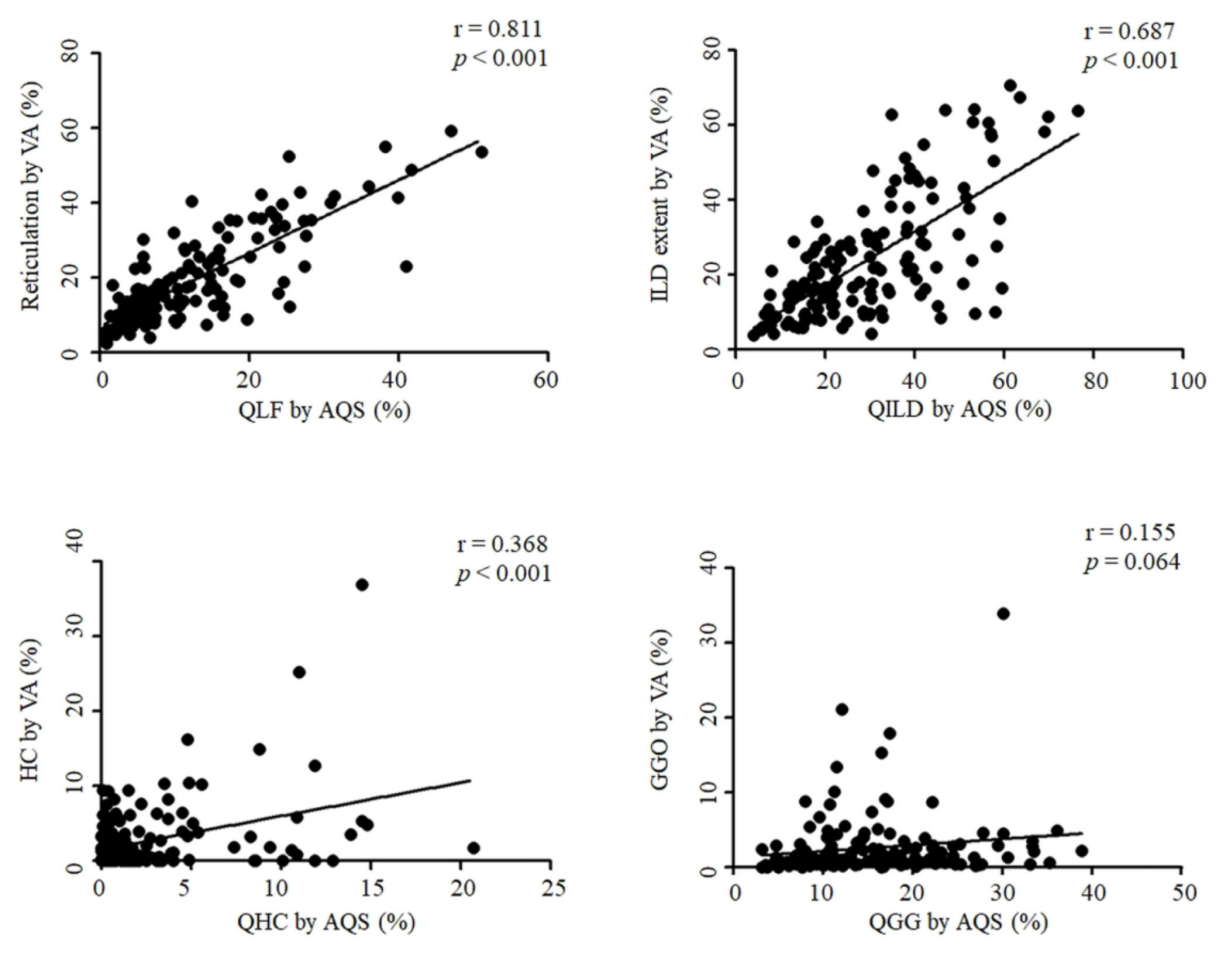
Correlation of high-resolution computed tomography scores between AQS and visual assessment VA, visual assessment; AQS, automated quantification system; HC, honeycombing; ILD, interstitial lung disease; GGO, ground-glass opacity; QLF, quantification of lung fibrosis; QILD, quantification of interstitial lung disease; QHC, quantification of honeycombing; QGG, quantification of ground-glass opacity

Non-survivors showed higher reticulation, honeycombing, and ILD extent scores, and more frequently had emphysema and a UIP pattern on visual assessment of HRCT images than survivors (Table S2). The QLF, QHC, QGG, and QILD scores on HRCT measured using the AQS were also higher in non-survivors than in survivors (Table 2). A significant positive correlation was found between HRCT scores measured through visual assessment and those measured using the AQS, except for GGO. The correlation was the highest for the reticulation score (r = 0.811, *P* < 0.001), followed by the ILD (r = 0.687, *P* < 0.001) and honeycombing (r = 0.368, *P* < 0.001) scores (Figure 2).

### Prediction of mortality

In the unadjusted Cox regression analysis, age, lung function (FVC, DLco, and TLC), ESR, presence of emphysema and UIP pattern on HRCT, and AQS scores (QLF, QHC, QGG, and QILD) were significantly associated with 5-year mortality in patients with RA-ILD (Supplementary Table S2, available at *Rheumatology* online). In the multivariable Cox analysis, higher QLF (hazard ratio [HR] 1.068, 95% confidence interval [CI] 1.026–1.113, *P* = 0.002), QHC (HR 1.090, 95% CI 1.021–1.164, *P* = 0.010), and QILD (HR 1.048, 95% CI 1.022–1.075, *P* < 0.001) scores were independent predictors of 5-year mortality along with older age and higher ESR (Table 3).

**Figure 3:**
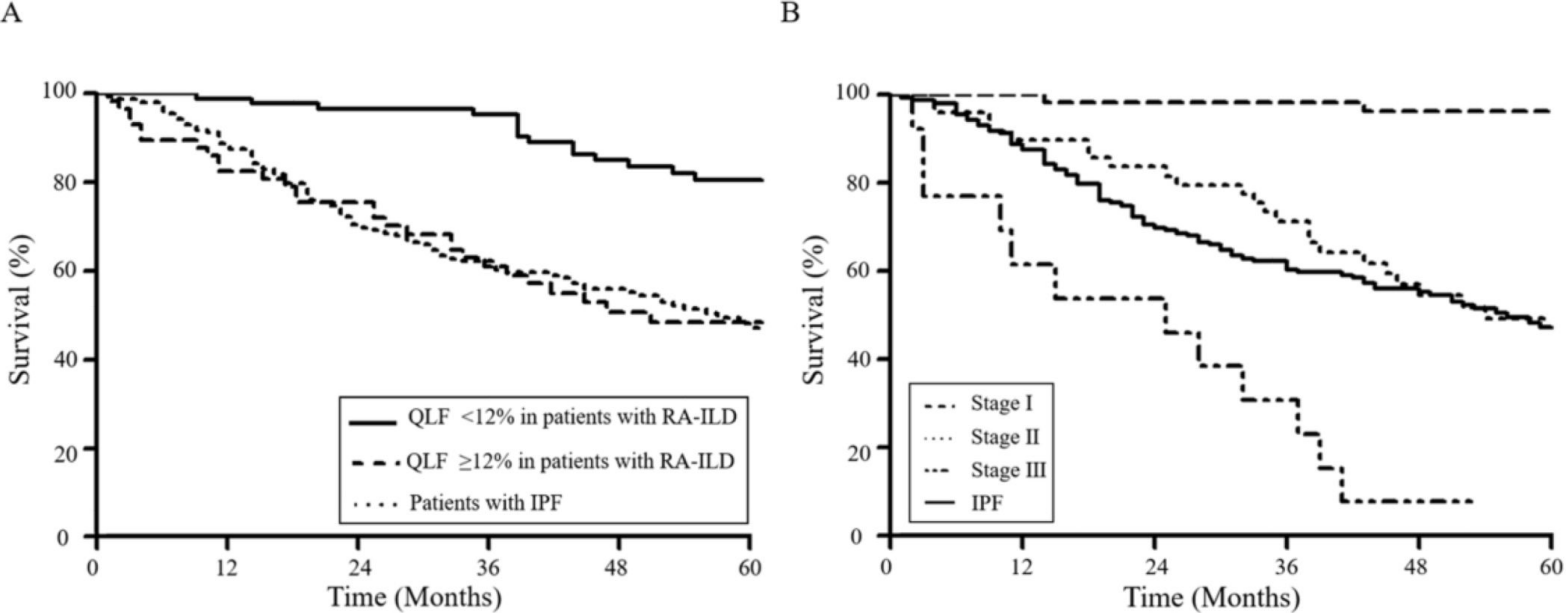
Comparison of Kaplan–Meier survival curves between patients with RA-ILD and patients with IPF A. Comparison of Kaplan–Meier survival curves between patients with RA-ILD subdivided according to the QLF score and patients with IPF, B. Comparison of Kaplan–Meier survival curves between patients with RA-ILD subdivided according to stage and patients with IPF Based on the points of variables including QLF, age and ESR, the patients were divided into 3 stages. Survival rates were significantly different at each stage and patients on stage II showed similar survival rate with those with IPF. RA-ILD, rheumatoid arthritis–associated interstitial lung disease; IPF, idiopathic pulmonary fibrosis; QLF, quantification of lung fibrosis

In the ROC curve analysis, the QLF and QILD scores were useful in predicting 5-year mortality and showed better performance (area under the curve 0.721 [QLF] vs. 0.744 [QILD], *P* = 0.285) than the QHC and QGG scores (Figure 1B). The optimal cut-off value of QLF was 12% (sensitivity 65.9%, specificity 71.0%). Patients with a high QLF score (≥ 12%, n = 57) showed higher 5-year mortality (50% vs. 17.4%, *P* < 0.001) than those with a low QLF score (< 12%, n = 87). Moreover, the survival outcome of patients with a high QLF score was similar to that of patients with IPF (Figure 3A).

### Combination with clinical variables

To improve the predictive performance of the QLF score in patients with RA-ILD, various models combined with clinical variables were compared (Supplementary Table S3, available at *Rheumatology* online). Among the prediction models, the model including the QLF score, age, and ESR showed better performance in predicting 5-year mortality (C-index 0.816 vs. 0.721, *P* = 0.017) than the model including the QLF score alone in patients with RA-ILD.

Based on the results of the ROC curve analysis for the optimal cut-off values (QLF score = 12%, age = 50 years, and ESR = 55 mL/dL) for 5-year mortality, continuous variables including age, ESR, and QLF score were converted into categorical variables. Points ranging from 0 to 2 were assigned to each variable based on the coefficient values (Supplementary Table S4, available at *Rheumatology* online), and patients were categorised into three stages according to the total points (range, 0–5) that demonstrated a similar 5-year survival rate (Supplementary Figure S1 and Supplementary Table S5, available at *Rheumatology* online). To reduce the overfitting bias of our model, bootstrap was performed 1000 times. Consequently, the 10% trimmed mean of the bootstrap-adjusted concordance was 0.749, and the 95% bootstrap confidence interval was found to be in the range of 0.692-0.806. This staging system including QLF scores and clinical variables demonstrated good separation for 5-year survival in patients with RA-ILD (*P* < 0.001) (Figure 3B).

## Discussion

This study revealed that HRCT scores measured using the AQS had a significant correlation with those measured through visual assessment and were effective in predicting 5-year mortality in patients with RA-ILD. The QLF score showed better performance than the QHC and QGG scores in predicting 5-year mortality and was helpful in distinguishing the group with a poor prognosis. Additionally, the results suggested that the combined model including the QLF score and clinical variables (age and ESR) may improve the performance of the QLF score in predicting 5-year mortality in patients with RA-ILD.

To overcome reader variability in the visual assessment of HRCT images, objective assessments using automated quantification methods have been attempted [12, 13, 25–28]. However, most previous AQS studies were conducted in patients with scleroderma-related ILD or IPF [11–14, 28–30]. Kim et al. analysed 129 patients with scleroderma-related ILD and observed a significant correlation (r = 0.60, *P* < 0.0001) between the QLF score measured using the AQS and the lung fibrosis scores (reticular pattern with architectural distortion) determined by two radiologists [12]. Our study showed similar findings in patients with RA-ILD in that the AQS scores were correlated with those obtained through visual assessment.

In this study, the QLF and QHC scores were independent prognostic factors for 5-year mortality in patients with RA-ILD. Some studies have evaluated the role of HRCT images in predicting survival in patients with RA-ILD [10, 27]. Jacob et al. investigated 157 patients with RA-ILD and reported that the reticulation and honeycombing extents assessed using the Computer-Aided Lung Informatics for Pathology Evaluation and Rating (CALIPER) software were both associated with mortality (HR 1.12, *P* < 0.001 and HR 1.17, *P* < 0.001, respectively, in the unadjusted Cox analysis) [27]. Nurmi et al. investigated 60 patients with RA-ILD and also showed that the extents of reticulation (HR 1.144, 95% CI 1.005–1.302, *P* = 0.041), traction bronchiectasis (HR 1.184, 95% CI 1.016–1.379, *P* = 0.030), and architectural distortion (HR 1.094, 95% CI 1.003–1.194, *P* = 0.044) on HRCT evaluated through visual assessment were associated with mortality in the univariate Cox analysis [10]. These findings were compatible with our results.

However, the QHC score showed a low correlation with the HC score in visual assessment. This discrepancy may have occurred because imaging findings that require differential diagnosis in determining HC, such as traction bronchiectasis, subpleural cysts or bulla and emphysema, cause some confusion in visual assessment and AQS analysis [31, 32]. In a previous study, it was found that there is high inter-observer variability (Cohen weighted κ values: 0.40-0.58) in CT evaluation of HC [32]. In addition, the QGG scores measured using the AQS were much higher than those measured using visual assessment, and they were not correlated with each other. These findings have also been identified in another previous study [33]. Marten et al. analysed 52 patients with connective tissue disease–associated ILD (including RA-ILD, n = 24) and showed that a high attenuation area (indicating the ILD extent) on HRCT measured using a computer-aided diagnostic tool (MeVisPULMO 3D software) was not correlated with the extent of GGO measured through visual assessment (r = 0.199, *P* = 0.199), in contrast to the results for the extents of ILD (r = 0.716, *P* < 0.0001) and reticulation (r = 0.690, *P* < 0.0001) [33]. The GGO score measured using the AQS may be overestimated because of atelectasis in the dependent portion of the lungs or decreased aeration area due to insufficient inspiration, whereas radiologists tend to underestimate the disease extent by considering image quality and noise level together when evaluating HRCT images [12].

In our study, older age and higher ESR were associated with poor prognosis in patients with RA-ILD. In previous studies, older age has been reported to be a poor prognostic factor for mortality in patients with RA-ILD [9, 27]. However, the association between ESR and mortality in patients with RA-ILD has been poorly defined. Previous studies suggested that ESR was associated with the development of ILD in patients with RA [34–36]. Koduri et al. investigated 1,460 patients with RA and reported that elevated ESR was associated with the development of ILD in multivariable Cox analysis (HR 1.01, 95% CI 1.00–1.02, *P* < 0.05) adjusting for age and health assessment questionnaire index [34]. Furthermore, Yang et al., in their study including 308 patients with RA, also reported that ESR was significantly higher (mean 47.9 ± 25.5 vs. 31.7 ± 21.9 mm/h, *P* = 0.022) in patients with ILD than in those without ILD. In addition, among patients with RA-ILD (n = 77), they found that non-survivors had higher ESR (58.0 ± 25.0 vs. 42.2 ± 24.3 mm/h, *P* = 0.008) than survivors [35]. These results are compatible with our findings. Moreover, ESR has also been reported to be correlated with the RA disease activity [37], which is a known risk factor for RA-ILD development [38].

This study had some limitations. First, this study was conducted at a single centre and had a retrospective design, which may limit the generalizability of our findings. However, the baseline characteristics of our patients were similar to those of patients in previous studies [5, 27, 39]. Second, the treatment was not considered in our model. Although no specific treatments have been proven effective for RA-ILD [40], treatment with steroidal and cytotoxic agents was not associated with survival in our study. Third, UIP pattern and emphysema, known as significant prognostic factors, were excluded in the multivariable analysis, although they showed significant values in the unadjusted analysis for predicting 5-year mortality. However, in our analysis, we intended to show the results with the exclusion of visual components to confirm the usefulness of the AQS. Finally, we did not include an external validation cohort to confirm the usefulness of the AQS. Therefore, the results need to be validated in another cohort. Nevertheless, comparisons with visual assessment showed that the AQS is a reliable tool for measuring the fibrosis extent, and previous studies based on visual assessment have shown that the fibrosis score is a significant prognostic factor in patients with RA-ILD [6, 8, 10]. Moreover, the IPF cohort was included as a control group for survival comparison in this study. In addition, the bootstrapping analysis for predictive model was performed for the internal validation. Despite these limitations, our study is valuable in that it demonstrated the reliability of AQS scores and their usefulness as independent prognostic factors in patients with RA-ILD even in consideration of clinical variables.

In conclusion, our results suggest that the QLF score might be useful in predicting prognosis in patients with RA-ILD, and high QLF scores may differentiate a poor prognostic phenotype.

## Supplementary Material

Figures

## Figures and Tables

**Table 1:** Comparison of baseline characteristics between non-survivors and survivors among patients with RA-ILD

Characteristics	Total	Non-survivors	Survivors	*P*-value
No. of patients	144	44	100	
Age, years	61.2 ± 10.1	65.0 ± 9.0	60.0 ± 10.2	0.003
Male sex	63 (43.8)	23 (52.3)	40 (40.0)	0.171
Ever-smokers	63 (43.8)	23 (52.3)	40 (40.0)	0.017
C-reactive protein, mg/dL	2.35 ± 4.2	3.1 ± 4.5	2.0 ± 4.1	0.139
ESR, mL/dL (n=119)	48.6 ± 31.3	64.6 ± 33.7	41.4 ± 27.4	< 0.001
RF positivity, %	109 (77.9)	36 (85.7)	73 (74.5)	0.143
RF titre, IU/mL	461.0 ± 980.7	820.0 ± 1460.2	303.5 ± 618.7	0.030
Anti-CCP positivity	112 (77.8)	47 (85.5)	65 (84.4)	0.870
Pulmonary function test				
FVC, % predicted	75.5 ± 18.8	68.4 ± 20.2	78.2 ± 17.4	0.003
DLco, % predicted	60.5 ± 16.6	52.9 ± 20.2	63.8 ± 18.6	0.002
TLC, % predicted	76.7 ± 16.6	71.7 ± 17.2	79.0 ± 15.9	0.017
Steroid ± IM	115 (79.9)	35 (79.5)	80 (80.0)	0.950
DMARDs	105 (72.9)	30 (68.2)	75 (75.0)	0.396
Biologics^*^	12 (8.3)	3 (6.8)	9 (9.0)	0.756

Data are presented as mean ± standard deviation or number (%), unless otherwise indicated. RA-ILD, rheumatoid arthritis–associated interstitial lung disease; ESR, erythrocyte sedimentation rate; RF, rheumatoid factor; CCP, cyclic citrullinated peptide; FVC, forced vital capacity; DLco, diffusing capacity of the lung for carbon monoxide; TLC, total lung capacity; IM, immunosuppressant (azathioprine, mycophenolate mofetil, cyclosporine; n = 50), DMARDs, disease-modifying antirheumatic drugs (methotrexate, leflunomide, calcineurin inhibitor, sulfasalazine, hydroxychloroquine);

* TNF a-inhibitors, rituximab and IL-6 inhibitors

**Table 2:** Comparison of HRCT scores between non-survivors and survivors among patients with RA-ILD

	Total	Non-survivors	Survivors	*P* - value
No. of patients	144	44	100	
Visual assessment scores				
Reticulation, %	19.3 ± 12.2	26.7 ± 13.4	16.1 ± 10.1	< 0.001
Honeycombing, %	2.7 ± 4.7	5.5 ± 6.3	1.5 ± 3.2	< 0.001
GGO, %	2.6 ± 4.2	3.0 ± 5.2	2.4 ± 3.7	0.435
Consolidation, %	0.4 ± 1.1	0.7 ± 1.2	0.3 ± 1.0	0.147
ILD extent^*^, %	24.6 ± 16.5	35.2 ± 17.2	20.0 ± 13.9	< 0.001
Emphysema	70 (48.6)	32 (72.7)	38 (38.0)	< 0.001
UIP pattern	53 (36.8)	26 (59.1)	27 (27.0)	< 0.001
AQS scores				
QLF, %	12.5 ± 10.1	17.9 ± 11.8	10.1 ± 8.3	< 0.001
QHC, %	2.7 ± 3.9	4.2 ± 4.5	2.0 ± 3.4	0.006
QGG, %	15.0 ± 8.0	17.5 ± 7.5	14.0 ± 8.0	0.013
QILD, %	30.3 ± 15.9	39.7 ± 15.7	26.2 ± 14.2	< 0.001

Data are presented as mean ± standard deviation or number (%), unless otherwise indicated. HRCT, high-resolution computed tomography; RA-ILD, rheumatoid arthritis–associated interstitial lung disease; GGO, ground-glass opacity; ILD, interstitial lung disease; UIP, usual interstitial pneumonia; AQS, automated quantification system; QLF, quantification of lung fibrosis; QHC, quantification of honeycombing; QGG, quantification of ground-glass opacity; QILD, quantification of interstitial lung disease;

* ILD extent was defined as the sum of reticulation, honeycombing, and GGO.

**Table 3:** Risk factors for 5-year mortality in patients with RA-ILD assessed using a multivariable Cox proportional hazards model

	HR (95% CI)			
Parameter	Model 1	Model 2	Model 3	Model 4
Age	1.059* (1.013–1.106)	1.050* (1.008–1.095)	1.052* (1.009–1.097)	1.047* (1.003–1.094)
ESR	1.015* (1.004–1.026)	1.012* (1.002–1.022)	1.012* (1.002–1.023)	1.015* (1.004–1.026)
FVC	0.984 (0.955–1.014)	0.974 (0.946–1.004)	0.988 (0.958–1.019)	0.992 (0.964–1.021)
DLco	1.005 (0.977–1.034)	0.989 (0.966–1.013)	0.987 (0.963–1.011)	0.999 (0.976–1.024)
AQS scores, % QLF	1.068* (1.026–1.113)			
QHC		1.090* (1.021–1.164)		
QGG			1.030 (0.983–1.079)	
QILD				1.048* (1.022–1.075)

TLC was not included in the multivariable analysis owing to its high correlation with FVC (r = 0.893, *p* < 0.001); RA-ILD, rheumatoid arthritis–associated interstitial lung disease; HR, hazard ratio; CI, confidence interval; ESR, erythrocyte sedimentation rate; FVC, forced vital capacity; DLco, diffusing capacity of the lung for carbon monoxide; AQS, automated quantification system; QLF, quantification of lung fibrosis; QHC, quantification of honeycombing; QGG, quantification of ground-glass opacity; QILD, quantification of interstitial lung disease; * *P* < 0.05.

## Key messages

- Quantitative lung fibrosis (QLF) scores might be useful in predicting prognosis in patients with RA-ILD.
- High QLF scores differentiate a poor prognostic phenotype of RA-ILD similar to IPF.
- Combining with clinical variables improve the performance of QLF score in predicting the mortality in patients with RA-ILD.
