# Supplementary material for: Automated quantification system predicts survival in rheumatoid arthritis–associated interstitial lung disease
Source: Rheumatology (Oxford). Author manuscript; Available in PMC 2023 Oct 6. (PMC7615169; doi:10.1093/rheumatology/keac184)

**Supplementary Information**

**Automated quantification system predict survival in rheumatoid arthritis–associated interstitial lung disease**

Ju Hyun Oh^1^, Grace Hyun J.Kim^2^, Gary Cross^3^, Joseph Barnett^3^, Joseph Jacob^4,5^, Seokchan Hong^6^, Jin Woo Song^1^

^1^ Department of Pulmonary and Critical Care Medicine, Asan Medical Center, University of Ulsan College of Medicine, Seoul, Republic of Korea

^2^ Department of Radiological Sciences, David Geffen School of Medicine at UCLA, Los Angeles, USA

^3^ Department of Radiology, Royal Free Hospital, Royal Free London NHS Foundation Trust, London, UK

^4^ Department of Respiratory Medicine, University College London, London, UK

^5^ Centre for Medical Image Computing, University College London, London, UK

^6^ Department of Rheumatology, Asan Medical Center, University of Ulsan College of Medicine, Seoul, Republic of Korea

Supplementary Table S1. Comparison of baseline characteristics between patients with RA-ILD and those with IPF

| Characteristics | RA-ILD | IPF | *P*-value |
| --- | --- | --- | --- |
| No. of patients | 144 | 159 |  |
| Age, years | 61.2 ± 10.1 | 67.0 ± 6.8 | < 0.001 |
| Male sex | 63 (43.8) | 130 (81.8) | < 0.001 |
| Pulmonary function test |  |  |  |
| FVC, % predicted | 73.7 ± 18.8 | 72.0 ± 18.2 | 0.470 |
| DLco, % predicted | 59.5 ± 19.1 | 57.1 ± 18.8 | 0.301 |
| Follow-up time, months | 52.5 (36.2–90.5) | 48 (21.0–59.0) | 0.001 |
| Non-survivors | 44 (30.6) | 80 (50.3) | < 0.001 |

Data are presented as mean ± standard deviation, median (interquartile range), or number (%), unless otherwise indicated.

RA-ILD, rheumatoid arthritis–associated interstitial lung disease; IPF, idiopathic pulmonary fibrosis; FVC, forced vital capacity; DLco, diffusing capacity of the lung for carbon monoxide

Supplementary Table S2. Risk factors for 5-year mortality in patients with RA-ILD assessed using an unadjusted Cox proportional hazards model

| Parameters | Hazard ratio | | 95% Confidence interval | *P* -value |
| --- | --- | --- | --- | --- |
| Age | | 1.051 | 1.017–1.086 | 0.003 |
| Male sex | | 1.572 | 0.870–2.842 | 0.134 |
| Ever-smokers | | 1.584 | 0.876-2.862 | 0.128 |
| C-reactive protein | | 1.039 | 0.981–1.101 | 0.189 |
| ESR | | 1.018 | 1.009–1.027 | < 0.001 |
| RF positivity | | 1.835 | 0.773–4.356 | 0.169 |
| Anti-CCP positivity | | 1.128 | 0.531–2.396 | 0.754 |
| FVC, % predicted | | 0.972 | 0.955–0.988 | 0.001 |
| DLco, % predicted | | 0.970 | 0.954–0.987 | 0.001 |
| TLC, % predicted | | 0.973 | 0.954–0.992 | 0.006 |
| Steroid ± IM | | 0.836 | 0.401–1.743 | 0.632 |
| DMARDs | | 0.670 | 0.355-1.265 | 0.217 |
| Biologics | | 0.662 | 0.205-2.139 | 0.491 |
| Emphysema | | 3.403 | 1.751-6.612 | <0.001 |
| UIP pattern | | 3.038 | 1.663–5.550 | < 0.001 |
| AQS scores, % | |  |  |  |
| QLF | | 1.063 | 1.039–1.089 | < 0.001 |
| QHC | | 1.088 | 1.030–1.148 | 0.002 |
| QGG | | 1.049 | 1.014–1.084 | 0.005 |
| QILD | | 1.049 | 1.030–1.067 | < 0.001 |

RA-ILD, rheumatoid arthritis–associated interstitial lung disease; ESR, erythrocyte sedimentation rate; RF, rheumatoid factor; CCP, cyclic citrullinated peptide; FVC, forced vital capacity; DLco, diffusing capacity of the lung for carbon monoxide; TLC, total lung capacity; IM, immunosuppressant (azathioprine, mycophenolate mofetil, cyclosporine; n = 50); DMARDs, disease-modifying antirheumatic drugs; UIP, usual interstitial pneumonia; HRCT, high-resolution computed tomography; AQS, automated quantification system; QLF, quantification of lung fibrosis; QHC, quantification of honeycombing; QGG, quantification of ground-glass opacity; QILD, quantification of interstitial lung disease

Supplementary Table S3. Comparison of the performance of risk prediction models for 5-year mortality in patients with RA-ILD

| Models | C-index | 95% CI | P-value |
| --- | --- | --- | --- |
| (1) QLF score | 0.721 | 0.633–0.809 | Reference |
| (2) QLF score + age | 0.761 | 0.676–0.845 | 0.202 |
| (3) QLF score + ESR | 0.794 | 0.710–0.863 | 0.058 |
| (4) QLF score + age + ESR | 0.816 | 0.733–0.899 | 0.017 |

RA-ILD, rheumatoid arthritis–associated interstitial lung disease; CI, confidence interval; QLF, quantification of lung fibrosis; ESR, erythrocyte sedimentation rate

Supplementary Table S4. Risk factors for 5-year mortality in patients with RA-ILD assessed using a multivariate Cox proportional hazards model

|  | Existing model | | | Bootstrapping sample model (n=1000) | | |
| --- | --- | --- | --- | --- | --- | --- |
| Variables | | HR (95% CI) | C-value | HR (95% CI) | C-value | Bias |
| QLF score > 12% | | 2.385  (1.348–4.221) | 0.869 | 2.369  (1.351-4.153) | 0.862 | 0.007 |
| Age > 50 years | | 5.023  (1.521–16.590) | 1.614 | 5.265  (1.284-18.433) | 1.582 | 0.032 |
| ESR > 55 mL/dL | | 5.353  (2.901–9.880) | 1.678 | 4.865  (2.807-9.875) | 1.661 | 0.017 |

RA-ILD, rheumatoid arthritis–associated interstitial lung disease; HR, hazard ratio; CI, confidence interval; C-value, Cox coefficient value; QLF, quantification of lung fibrosis; ESR, erythrocyte sedimentation rate

Supplementary Table S5. Points assigned to each variable and the staging system

| Variables | Category | Points |
| --- | --- | --- |
| QLF score > 12% | No | 0 |
|  | Yes | 1 |
| Age > 50 years | No | 0 |
|  | Yes | 2 |
| ESR > 55 mL/dL | No | 0 |
|  | Yes | 2 |
| Stage | Ⅰ | 0–2 |
|  | Ⅱ | 3–4 |
|  | Ⅲ | 5 |

QLF, quantification of lung fibrosis; ESR, erythrocyte sedimentation rate

**FIGURE LEGEND**

Figure S1. Five-year survival rate of patients with rheumatoid arthritis–associated interstitial lung disease according to total points


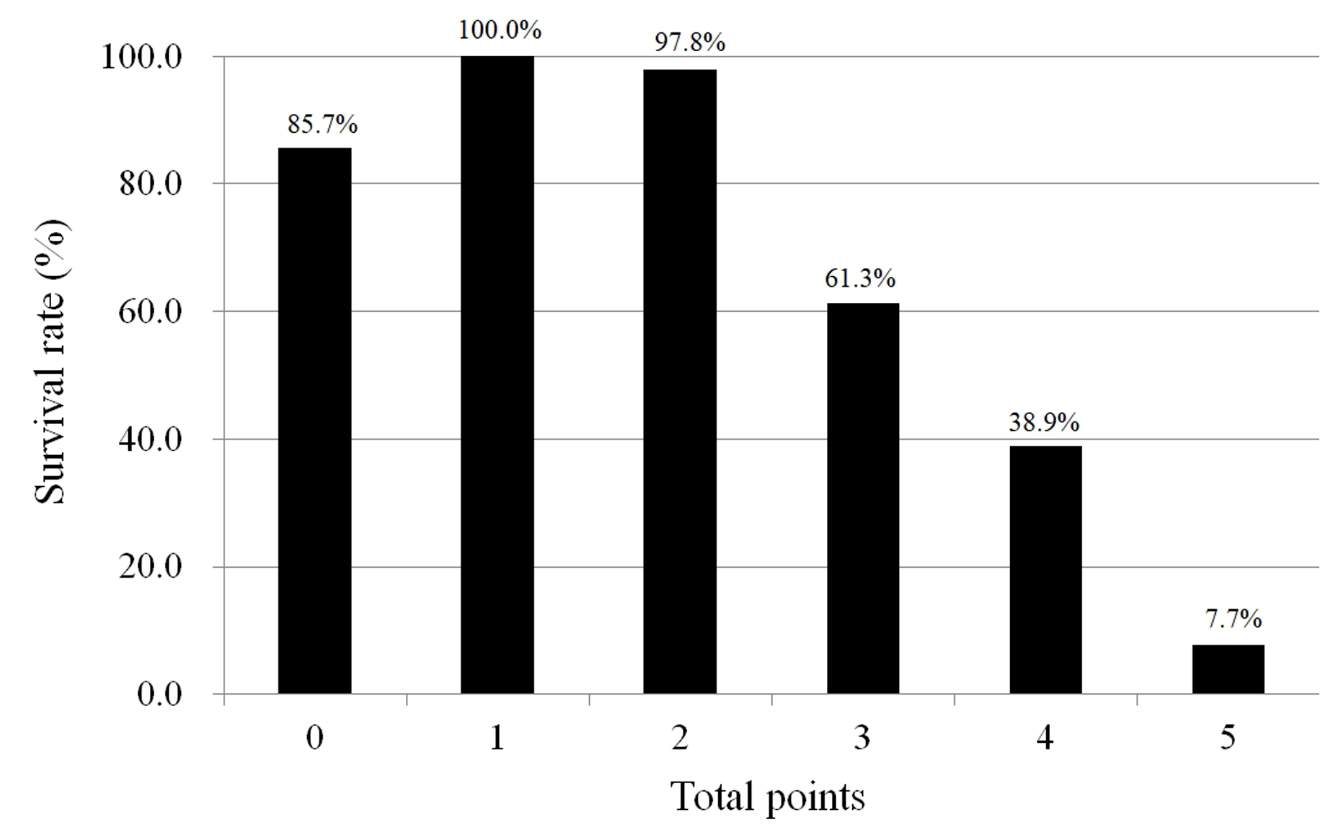

Supplement: Figures [file EMS188577-supplement-Figures.docx]
